# Comparative ovarian morphophysiology of *Wistar* rats and Zebrafish after exposure to nandrolone decanoate

**DOI:** 10.1590/1984-3143-AR2024-0046

**Published:** 2025-01-17

**Authors:** João Eudes Farias Cavalcante, Solano Dantas Martins, Jonathan Elias Rodrigues Martins, Jéssica Sales Lobato, Yara Silvino Sales, Sara Rany Alexandre Bittencourt, Fernanda Vitória Almeida Magalhães, Ana Ruth Reinaldo Menezes, Maria Alice Felipe Oliveira, Vânia Marilande Ceccatto, Anderson Weiny Barbalho Silva, Carminda Sandra Brito Salmito-Vanderley, Valdevane Rocha Araújo

**Affiliations:** 1 Programa de Pós-graduação em Ciências Veterinárias – PPGCV, Faculdade de Medicina Veterinária – FAVET, Universidade Estadual do Ceará – UECE, Fortaleza, CE, Brasil; 2 Laboratório de Biotecnologia da Reprodução de Peixes – LBRP, Núcleo Integrado de Biotecnologia – NIB, Faculdade de Medicina Veterinária – FAVET, Universidade Estadual do Ceará – UECE, Fortaleza, CE, Brasil; 3 Programa de Pós-graduação em Biotecnologia – PPGBiotec, Universidade Federal do Delta do Parnaíba – UFDPar, Parnaíba, PI, Brasil; 4 Programa de Pós-graduação em Biotecnologia – RENORBIO, Universidade Estadual do Ceará – UECE, Fortaleza, CE, Brasil; 5 Laboratório de Bioquímica e Expressão Gênica – LABIEX, Universidade Estadual do Ceará – UECE, Fortaleza, CE, Brasil; 6 Centro de Ciências da Saúde, Universidade Estadual do Ceará – UECE, Fortaleza, CE, Brasil; 7 Programa de Pós-graduação em Ciências Fisiológicas – PPGCF, Instituto Superior de Ciências Biomédicas – ISCB, Universidade Estadual do Ceará – UECE, Fortaleza, CE, Brasil; 8 Laboratório de Biotecnologia e Fisiologia da Reprodução – LABIREP, Núcleo de Pesquisa em Experimentação Animal – NUPEX, Programa de Pós-graduação em Biotecnologia, Universidade Federal do Ceará – UFC, Sobral, CE, Brasil; 9 Curso de Graduação em Ciências Biológicas, Universidade Federal do Delta do Parnaíba – UFDPar, Parnaíba, PI, Brasil

**Keywords:** ovarian follicles, anabolic steroids, cell culture, Danio rerio, Rattus norvegicus

## Abstract

This study aimed to compare the effects of nandrolone decanoate on the morphology and physiology of ovarian tissues in two experimental models, Zebrafish and rats, after in vitro cultivation. A total of 136 animals were used (*Wistar* rats, n=36, and Zebrafish, n=100). In both experiments, the animals were divided into two groups (Control and Deca) and were exposed to nandrolone decanoate for seven weeks. At the end of the administrations, the animals were euthanized, and the tissues were collected for morphological and biochemical analyses. Data were expressed as mean ± SEM. Tukey and Shapiro-Wilk tests were used. ANOVA and chi-square tests were applied for group comparisons. Differences were considered significant when P<0.05. The results showed an increase in body weight in *Wistar* rats, while Zebrafish body weight was decreased. In both experiments, the number of atretic follicles increased throughout the in vitro culture, from day 0 to day 7, in the Control group (CTRLr and CTRLz), while in the DECA group (DECAr and DECAz), atretic follicles were reduced from D0 to D7. The antioxidant environment, represented by increased the thiol content, which was significantly higher on day zero in CTRLz compared to CTRLr. SOD activity increased in Zebrafish (group DECAz), while CAT activity decreased in both models (group DECAr and DECAz). In conclusion, the study demonstrated similarity in ovarian physiology between the models exposed or not exposed to nandrolone decanoate, suggesting that, when convenient, researchers could consider changing the experimental model.

## Introduction

The Zebrafish has been a widely used model for evaluating new compounds in various fields, such as health safety (Bailone et al., 2020). Characteristics like transparency, external fertilization, small size, and short life cycle support faster in vivo assessments of new drugs, for example. Additionally, genetic homology with humans (70%) and low cost have been highlighted as the main advantages of using this model instead of mammalian models (Lee and Renshaw, 2017). Thus, the potential efficiency and practicality of this model could optimize the development process for researchers who routinely use mammalian models, such is rats. *Wistar* lineage (*Rattus norvegicus*) is one of the most used worldwide due to their small size, short biological cycle, low breeding cost, and degree of genetic similarity (80%) with humans. Such characteristics allow the extrapolation of results to estimate potential effects of a given treatment in humans (Mattaraia and Moura, 2012).

A research example that can be used is to evaluate the anabolic steroids effects. These compounds are similar to testosterone and are produced by organs, such as adrenal gland and gonads. Clinically, the synthetic formulation of anabolic steroids was developed to treat catabolic status in immunosuppressed patients (Shahidi, 2001). Nandrolone decanoate (DECA) is one of the most common synthetic anabolic-androgenic steroids (Boff, 2010; Simão et al., 2021). Its moderate androgenic potential and anabolic properties (Dutra et al., 2012; Patanè et al., 2020) made DECA the most synthetic anabolic popular. Although there is control over the prescription and commercialization of these drugs in many countries (Zaami et al., 2021), use occurs indiscriminately by adolescents and adults with the aim of increasing muscle mass and reduce subcutaneous fat (Pope and Katz, 1997; Iriart et al., 2009; Silva et al., 2015).

The recommended therapeutic dose of DECA for humans is 0.4 mg/kg/day (Tamaki et al., 2003). However, it has been used in supra-physiological doses because its androgenic and anabolic properties, reaching up to 100 times the therapeutic recommendation (Clark and Fast, 1996). It is important to highlight that the exacerbate use of the steroid anabolic results in numerous adverse effects, including damage to steroid-dependent organs, such as the female and male reproductive organs (Saddick, 2018; Gomes et al., 2020).

Effects on female reproduction have been reported in the last decade, including menstrual cycles disorders, oligomenorrhea, amenorrhea or anovulation (Cannavò et al., 2001). Moreover, also have being observed breast atrophy, clitoral hypertrophy (Hoffman and Ratamess, 2006), and histopathological changes in the ovary, uterus, and oviduct. All these alterations suppress female reproductive capacity (Belardin et al., 2014; Camargo et al., 2009b, 2014) in a dose- and time-dependent manner (Belardin et al., 2014; Simão et al., 2016; Andrade et al., 2018; Saddick, 2018; Itohan et al., 2020).

Considering the indiscriminate use of DECA in women, its effects on reproductive physiology still need to be further explored. Additionally, human biological samples are scarce and raise a series of ethical issues that complicate experimental studies. Alternatively, other biological models such as zebrafish and *Wistar* rats (Westerfield, 2007) can be used as substitutes for humans. Thus, the aim of the study was to compare zebrafish and *Wistar* rats as experimental models to evaluate ovarian physiology after exposure to DECA and recovery through in vitro culture.

## Methods

The experiments were carried out at the Laboratory of Biotechnology of Fish Reproduction (LBRP), part of the Integrated Center for Biotechnology – NIB/UECE, of the Faculty of Veterinary Medicine (FAVET) of the State University of Ceará (UECE – Fortaleza – CE – Brazil), as well as in the Laboratory of Biochemistry and Gene Expression (LaBiEx) and in the Laboratory of Cell Culture (LabCult) attached to LABIEX, both belonging to the Superior Institute of Biomedical Sciences (ISCB). This study was approved by the UECE Ethics Committee for the Use of Animals (CEUA/UECE n° 03041520/2021).

### Experimental animal models

A total of 136 animals (rats, *n*=36 and fish, *n*= 100) were used to both experiments. For Experiment 1, 36 Female *Wistar* rats with twenty-six weeks old and weight ranging from 170 to 200 g were obtained from ISCB/UECE Bioterium. The rats were housed in groups (*n*=5) in standard polyethylene cages (41 cm x 34 cm x 16 cm) lined with wood shavings, under controlled temperature (23-25 °C), and a 12 h light–dark cycle, with free access to food and water.

For Experiment 2, 100 Zebrafish were commercially acquired and housed in groups (*n*=10) in aquariums with eight liters of water, and photoperiod conditions of 14 hours of light and 10 hours of dark. The fish were fed once a day and the aquarium water partially changed twice a week before and after 42 h of DECA exposition. A sample from water change of each aquarium was used to measure the parameters of temperature, pH, and dissolved ammonia, nitrite, oxygen, and hardness. Temperature was measured using a thermometer, while the other parameters were checked by colorimetric tests (LabconTest) (Westerfield, 2007).

### Experimental protocol

Table 1 demonstrates the distribution of the experimental groups. Both animal species received 10 mg/kg of Nandrolone Decanoate (Deca Durabolin®-Aspen PHARMA) (DECA group) or its vehicle, i.e., peanut oil with 10% benzyl alcohol, and were designated as the Control (CTRL) group.

**Table 1:** Characterization of the experimental groups in the absence (Control group, Ctrl) and presence of a supraphysiological dose of 10 mg/kg of Nandrolone Decanoate (Deca group) in two experimental models.

**Experiment**	**Species**	**Group**	**Period**	**Animals (nº)**	**Analyzes (nº of ovaries)**	
					**CH**	**B**
Experiment 1	Rats	Ctrl-r in vivo	D0	08	08	08
		Ctrl-r *in vitro*	D1	05	05	05
			D7	05	05	05
		Deca-r in vivo	D0	08	08	08
		Deca-r *in vitro*	D1	05	05	05
			D7	05	05	05
Experiment 2	Zebrafish	Ctrl-z in vivo	D0	10	10	10
		Ctrl-z *in vitro*	D1	20	20	20
			D7	20	20	20
		Deca-r in vivo	D0	10	10	10
		Deca-z *in vitro*	D1	20	20	20
			D7	20	20	20
Total de animais (n°):				136		

Ctrl-r: Rats Control Group; Deca-r: Deca-Rats Group; Ctrl-z: Zebrafish Control Group; Deca-z: Deca Zebrafish group; CH: Classical histology; B: Biochemistry; D0/D1/D7: Culture period.

Experiment 1 was conducted to evaluate whether in vivo administration of DECA would be reversed after in vitro culture of murine ovarian tissue. For this, the female rats were treated for 7 weeks via intramuscular injection (CTRLr group, n=18 and DECAr group, n=18), mimicking chronic exposure to the drug, as previously described by Alves et al. (2023). A single weekly intramuscular injection at a dose of 10 mg/kg of nandrolone decanoate (Deca Durabolin® Organon, SP) was administered (Karbalay-Doust et al., 2007). The Deca Durabolin from Organon do Brazil (São Paulo, Brazil) was bought on local pharm with medical prescription and the peanut oil was obtained on local commerce. Each ampule Deca Durabolin contained 1 mL oily solution. The peanut oil has 10% of benzoic alcohol on your composition.

At the end of the administrations, the animals were euthanized 48 hours later, and the tissues were collected and weighed. Part of the ovaries (n=16) was used for morphological and biochemical analyses of the fresh tissue, which constituted an in vivo control group or Day 0 (D0), while the other part (n=20) was cultured in vitro for 1 or 7 days (D1; n=10 and D7, n=10 ovaries). Subsequently, these ovaries were also subjected to the same analyses mentioned for D0.

For Experiment 2, the same endpoints of Experiment 1 were performed in the Zebrafish ovaries. Just like for Experiment 1, the animals were exposed to DECA, in this case, for 42 hours (DECAz group, *n*=50). Towards, animals from five aquariums were housed into other five aquariums (10 animals each) containing four liters of water, to which was added 10 mg/kg of DECA rediluted in alcohol (95 °GL, 0.04%) and then covered with a dark film. After the exposition, animals returned to their original aquarium staying there until next week i.e., until next exposition.

To guarantee the same stress process as DECAz group, the CTRLz group was composed by animals (*n*=50) that were housed into five aquariums (10 animals each) containing four liters of water, without DECA exposition, but also during 42 h, and under a dark film. After that, animals from CTRLz group also returned to their original aquarium staying there until next week. At the end of the experimental time (seven weeks), and after euthanasia, ovaries of all animals from both groups (CTRLz and DECAz) were dissected and immediately stored to biochemistry and morphologically analyzes (20 ovaries, named as *in vivo* control or D0) or forwarded to *in vitro* culture for 1 or 7 days (D1; n=40 and D7, *n*=40 ovaries). After the *in vitro* culture period, ovaries were destined to the same analysis cited previously.

### Euthanized and tissue collection

At the end of the experiments, after 48 (Experiment 1 – *Wistar* rats) or 42 h (Experiment 2 – Zebrafish) of DECA exposition, animals were euthanized. Female rats were anesthetized (ketamine 60 mg/Kg e xylazine 8 mg/Kg via intraperitoneal) and then killed by decapitation with a guillotine. On the other hand, the female Zebrafish were euthanized by clover oil immersion (eugenol) at 1:9:2500 (eugenol: alcohol: water).

### Estrous cycle evaluation

The estrous cycle was evaluated only for Experiment 1. Once a week, concomitant to administration of DECA, female rats were monitored to determine the phase of the estrous cycle (diestrus, proestrus, estrous, metestrus), as described previously (Marcondes et al., 2002; Goldman et al., 2007; Furtado et al., 2021). For that, 20 µL of saline solution 0.9% were injected to vagina channel and up and down pipetting three to four times. Forwards, the vaginal cell suspension was analyzing by optical microscopy (20x, Nikon Eclipse E100®, Japan) to identify round and nucleated epithelial cells (proestrus), irregular or misshaping anucleated keratinized cells, also known as cornified cells (estrous), or predominance of little round cells leucocyte, which are the leukocytes cells (diestrus).

### Morphological analysis and assessment of quality of ovarian follicle

Before (D0) and after one (D1) or seven days of culture (D7), ovaries were fixed in 4% paraformaldehyde for 4 hours and then stored in alcohol 70% until histological process. Summary, the ovaries were dehydrated in increasing concentrations of ethanol and diaphanized in xylene. Next, ovaries were embedded in paraffin (Synth, São Paulo, Brazil), and sectioned (7 µm). The ovary sections were mounted on glass slides and either stained with haematoxylin-eosin for histological analysis. Follicle stage and quality were assessed under a light microscope (40x, Nikon Eclipse E100, Japan®) using coded anonymized slides.

**Figure 1:**
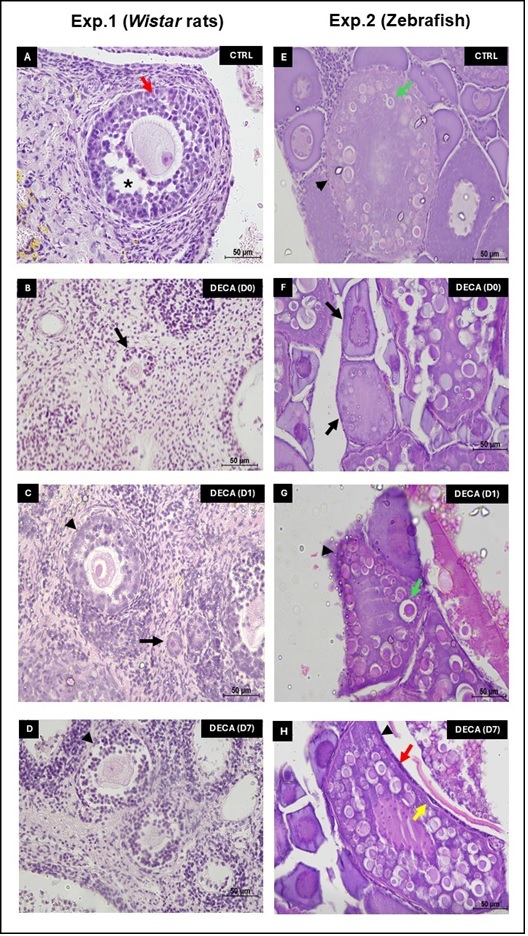
Ovarian follicles from *Wistar* rats (Experiment 1, A-D) and Zebrafish (Experiment 2, E-H) after 7 weeks of treatment using only vehicle composed by peanut oil (CTRL group, A and E) or 10 mg/kg of Nandrolone decanoate (DECA group, B, C, D, F, G and H) before (B and F) and after 1 (C and G) or 7 days (D and H) of in vitro culture. Antral (A) normal follicle on day 0 from Experiment 1 (CTRLr). Primary (B) and Secondary (C and D) degenerated follicles after DECA exposure before (B) and after one (C) or seven (D) days of in vitro culture from Experiment 1 (DECAr). Vitelogenic (E) normal follicle on day 0 from Experiment 2 (CTRLz). Cortical alveolus (F) and vitelogenic (G-H) degenerated follicles after DECA exposure before (F) and after one (G) or seven (H) days of in vitro culture from Experiment 2 (DECAz). Asterisk represents antral cavity from rat antral follicle. Arrow represents rat primary follicle or zebrafish cortical alveolus follicle. Red arrow represents the granulosa cells. Yellow arrow represents the yolk envelope. Green arrow represents the cortical alveoli. Arrowhead represents rat secondary follicle or zebrafish vielogenic follicle.

For experiment 1, the *Wistar* rats ovarian follicles were classified in primordial as quiescent follicles, and primary, secondary and tertiary as development follicles (Araújo et al., 2014). Primordial follicles were characterized by presence of a one single layer of flattened granulosa cells around the oocyte). Primary follicles were characterized by a single layer of cuboidal granulosa cells around the oocyte. Secondary follicles were characterized by oocyte surrounded by two or more layers of cuboidal granulosa cells without antral cavity. Finally, tertiary or initial antral follicles were characterized several granulosa cells layers and by the presence of antral cavity among granulosa cells. Follicles were classified individually as histologically normal when an intact oocyte was present, i.e., an oocyte without a pyknotic nucleus or cytoplasmic retraction, surrounded by granulosa cells, well organized in one or more layers and that have no pyknotic nucleus (Exp. 1; Figure 1A). On the other hand, atretic follicles were defined as those with a retracted oocyte, pyknotic nucleus, and/or disorganized granulosa cells detached from the basement membrane (Exp. 1; Figure 1B-D) (Hulshof et al., 1994; Araújo et al., 2010). Overall, at least, 150 follicles were evaluated for each treatment (30 follicles/group/animal). The percentages of follicles were calculated before (D0) and after in vitro culture (D1 and D7). The percentages of normal or atretic follicles were calculated by the number of morphological normal follicles or the number of atretic follicles, respectively, in relation to the total number of the follicles x100. The percentage of primordial follicles was calculated based on the number of normal primordial follicles divided by the total of morphological normal follicles x100. Regarding the percentage of development follicles, it was calculated summing the number of primary, secondary and tertiary follicles, divided by the total of normal follicles x100.

For experiment 2, Zebrafish follicles were classified into primordial as quiescent follicles, and cortical alveolus, vitellogenic, and mature follicles as development follicles (Selman et al., 1993). Primordial follicles were characterized by an oocyte surrounded by few cells; while the cortical alveolus follicles were those with oocytes distinguished by the formation of cortical alveoli, and presence of cuboidal follicular cells around the oocyte. Already vitellogenic follicles were characterized by the vitelline envelope and oocyte growth; while the mature follicle presented theca and granulosa cells, and presence of yolk organized around the oocyte. Fish follicles were classified individually as histologically normal when an intact oocyte was present surrounded by granulosa cells well organized in one or more layers around the oocyte, presence of the organized cortical alveoli, and/or vitelline envelope, and/or yolk (Exp. 2; Figure 1E) (Selman et al., 1993). On the other hand, atretic follicles were defined as those with disorganized granulosa cells detached from the basement membrane, retraction and invagination of the zona radiata, hyperplasia in cortical alveoli and basal membrane disintegration (Exp. 2; Figure 1F-H) (Migliaccio et al., 2018). As for Experiment 1, the Zebrafish 150 follicles were counted and the percentages were calculated before (D0) and after culture (D1 and D7).

### Total proteins (Bradford method)

The protein concentration was determined using the Bradford method (Bradford, 1976). This method uses Coomassie blue (Quick start/Bradford; Catalogue No. 500-0205; Bio-Rad) to determine the total concentration of proteins in each extract sample. When it comes in contact with proteins, the Coomassie blue stain forms a complex and emits a blue luminescence. The absorbance is directly related to the protein concentration of the sample and was evaluated spectrophotometrically at a wavelength of 595 nm. The total protein concentration in samples was determined using a standard curve of bovine albumin (0, 2.5, 5, 10, 15, 25, 35 and 50 mg/mL), which was used to standardize the levels of pro-oxidants (thiol) and antioxidants (SOD, CAT), as described below.

### Determination of pro-oxidant based on the thiol content

Total thiol content was determined using 5,5`-dithiobis 2-nitrobenzoic acid (DTNB; D8130; Sigma-Aldrich Chem. Co, St Louis, USA) as an index of reduced thiol molecules. Thiol residues react with DTNB (10 mM), cleaving the disulfide bond to form 2-nitro-5-thiobenzoate anion (NTB2–) at a neutral pH. NTB2– is quantified in a spectrophotometer by measuring absorbance at 412 nm, with results expressed as nMol of reduced DTNB per milligram of protein (Furtado et al., 2021; Takahashi et al., 1978).

### Antioxidant enzyme activities (SOD and CAT)

Each 100 mg of ovarian tissue was homogenized 3 to 4 times, for 10 seconds at 4 °C, in 900 μL of buffer (10mM Tris-HCl, 0.9% NaCl (w/v), pH 7.4), using an Ultra-turrax homogenizer T25. The samples were centrifuged for 10 min at 4 °C and 720 G of speed. The supernatant was used to test the activity of the enzymes specified below, after determining the protein content of the sample using the Bradford method (Furtado et al., 2021; Bradford, 1976).

#### Superoxide dismutase (SOD) activity

SOD activity was measured as the inhibition of adrenaline auto-oxidation (Bannister and Calabrese, 1987). Adrenaline oxidation, in the presence of CAT in basic medium, leads to the formation of the – O2^–^ radical, which SOD reacts with, thus slowing (‘inhibiting’) the oxidation of adrenaline. The CAT solution (0.048 mg/mL; c9322; Sigma-Aldrich Chem. Co St Louis, USA) was performed adding (7:3) to glycine buffer, pH 10.2 (Dinâmica Química, São Paulo, Brazil). Three different volumes (10, 20 or 40 μL) of ovary homogenate were then added to the solution and then adrenaline (0.218 mg/mL; E4260; Sigma-Aldrich Chem. Co, St Louis, USA) was added to start oxidation. Oxidation was measured at 480 nm every 10 s for 180 s.

#### Catalase activity (CAT)

CAT activity was measured as the consumption of H2O2 as a substrate at 240 nm (Aebi, 1984). A solution of H_2_O_2_ (152 µL/mL; PH09717RA; Êxodo Científica, Sumaré, Brazil) and phosphate-buffered saline (PBS; pH7.4) was mixed in a quartz cuvette at room temperature, and then 50 μL of the ovary homogenate were added. Every 30 seconds, the consumption of H_2_O_2_ was measured twice.

### *In vitro* culture of ovarian tissue

After euthanasia, the ovaries were dissected and washed once in 70% alcohol for 10 seconds and twice in saline supplemented with 100 µg/mL of penicillin and 100 µg/mL of streptomycin and sent to the laboratory at 4 °C. In the laboratory, adipose tissue and surrounding ligaments were removed and the ovaries were individually cultured in a 24-well culture dishes containing 1 mL of culture media. For both animal species, *in vitro* culture was performed at 37 °C in 5% CO^2^ in a humidified incubator. The basic culture medium consisted of αMEM (pH 7.2-7.4) supplemented with ITS (10 μg/mL insulin, 5.5 μg/mL transferrin, and 5 ng/mL selenium), 2 mM glutamine, 2 mM hypoxanthine, 1.25 mg/mL of bovine serum albumin (BSA). The total change of medium was performed every 2 days (Araújo et al., 2010).

### Statistical analysis

Unless indicated otherwise, results were expressed as the mean ± s.e.m. and as a frequency observation percentage. Data were initially evaluated for homoscedasticity and normality using Tukey and Shapiro–Wilk tests. Comparisons among groups were made using two-way analysis of variance (ANOVA) and qui-square. Differences were considered significant at *P*<0.05 using GraphPad Prism 7.0 (GraphPad Software®).

## Results

### Characterization of animal models and their samples

#### Body weight from *Wistar* rats (Exp. 1) and Zebrafish (Exp. 2)

**Figure 2:**
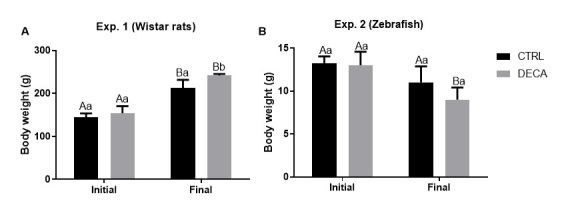
Initial and final body weight (g) of female animals of (A) *Wistar* rats (Exp. 1) and (B) Zebrafish species (Exp. 2) after 7 weeks of treatment using only vehicle composed by peanut oil (CTRL group) or 10 mg/kg of nandrolone decanoate (DECA group). Values are expressed in mean ± s.e.m. Control (CTRL) groups, *Wistar* rats (CTRLr) and Zebrafish (CTRLz) without DECA exposition. DECA.groups, *Wistar* rats (DECAr) and Zebrafish (DECAz) exposed to Nandrolone decanoate (DECA) for 42 hours. ^A,B^Indicate difference between initial and final body weight at CTRL or DECA group within the same animal species, i.e., rat (CTRLr initial x CTRLr final or DECAr initial x DECAr final) or zebrafish (CTRLz initial x CTRLz final or DECAz initial x DECAz final) (*P*<0.05). ^a,b^Indicate difference between CTRL and DECA group within the same experimental period, i.e., initial or final (CTRLr x DECAr or CTRLz x DECAz) period (*P*<0.05).

Figure 2 demonstrates the initial and final body weight of female rats (Exp. 1; Figure 2A) and Zebrafish (Exp. 2; Figure 2B) after 7 weeks of treatment using only vehicle composed by peanut oil (CTRL group) or 10 mg/kg of nandrolone decanoate (DECA). For Experiment 1, the body weight increased significantly (P<0.05) in both groups (CTRLr and DECAr) after 7 weeks, being DECAr group higher (P<0.05) then CTRLr group. In Experiment 2, the DECAz group significantly decreased (P<0.05) body weight after 7 weeks of treatment when compared to CTRLz.

### Estrous cycle assessment from female *Wistar* rats (Exp. 1)

**Figure 3:**
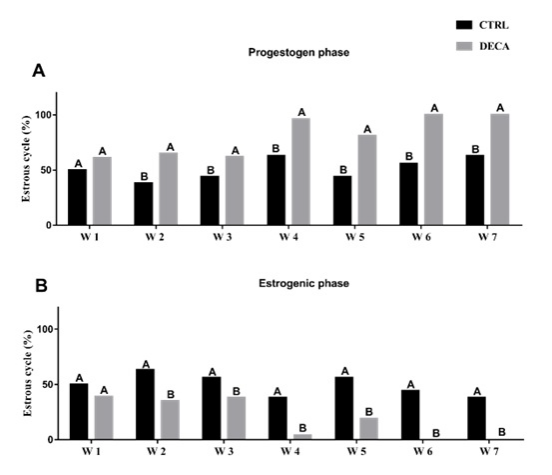
Estrous cycle phases (%) in (A) progestogen phase and (B) estrogenic phase of female animals of rat (Experiment 1) after 7 weeks of treatment using only vehicle composed by peanut oil (CTRL group) or 10 mg/kg of Nandrolone decanoate (DECA group). Values are expressed in mean ± s.e.m. ^A,B^Indicate difference between of CTRL or DECA in the same week (W) in females *Wistar* rats, i.e., *Wistar* rats in (A) progestongen phase (CTRLr W 1 x DECArW 1 or CTRLr W 2 x DECAr W 2 or CTRLr W 3 x DECAr W 3 or CTRLr W 4 x DECAr W 4 or CTRLr W 5 x DECAr W 5 or CTRLr W 6 x DECAr W 6 or CTRLr W 7 x DECAr W 7) or (B) estrogenic phase (CTRLr W 1 x DECAr W 1 or CTRLr W 2 x DECAr W 2 or CTRLr W 3 x DECAr W 3 or CTRLr W 4 x DECAr W 4 or CTRLr W 5 x DECAr W 5 or CTRLr W 6 x DECAr W 6 or CTRLr W 7 x DECAr W 7) (*P*<0.05).

The monitoring of estrous cycle was performed during the role experimental time, i.e., during the 7 weeks for both groups of *Wistar* rats from Experiment 1. Figure 3 shows that, in both groups (CTRLr and DECAr) female rats cycled during the first week of exposure, transitioning between the estrogenic phase (61%) and the progestogen phase (39%) (P<0.05). From the second week onward (65%), treatment with DECA disrupted the estrous cycle, keeping the animals in the progestogen phase (P<0.05). This means that by the seventh week, 100% of the female *Wistar* rats in the DECAr group were in diestrus (P<0.05). In the control group (CTRLr), the animals started with half of the population in the estrogenic phase and remained with at least 40% of the animals transitioning between the proestrus or estrus phases (P<0.05).

### Water quality parameters from aquarium water change of Zebrafish (Exp. 2)

The water quality can be observed at Table 2. Parameters of temperature, pH, and dissolved oxygen, and where similar between groups (CTRLz vs DECAz), being in according to the parameters considered appropriate. On the other hand, the dissolved ammonia and nitrite was reset and oxygen decreased (4-8 mg/L) close to the minimum reference value (4 mg/L) after DECA exposition (DECAz group).

**Table 2:** Water quality parameters of temperature, pH, and dissolved ammonia, nitrite, oxygen, and hardness from aquarium water change of Zebrafish (Exp. 2) before and after 42 h of exposition to 10 mg/kg of Nandrolone decanoate (DECAz).

**Endpoint**	**Before exposition**		**After exposition**		**Reference Value**
	**CTRLz**	**DECAz**	**CTRLz**	**DECAz**	
Temperature (^o^C)	26-28	26-28	26-28	26-28	24-28
pH (ppm)	7.2-7.6	7.2-7.6	7.2-7.6	7.2-7.6	6.8-8.5
NH_3_ (ppm)	0.007	0.007	0.007	0	0
NH_2_ (ppm)	0.25	0.25	0.25	0	0
O_2_ (mg/L)	6-11	6-11	6-11	4-8	>4
Hardness (mg/L)	80-170	80-170	80-170	80-170	75-200

Control (CTRLz) group, Zebrafish without DECA exposition, but under a dark film also during 42 h. DECAz group, Zebrafish exposed to Nandrolone decanoate (DECA) for 42 hours. NH_3_, Dissolved ammonium, NH_2_, nitrite, and O_2_, oxygen.

### Morphological quality of *Wistar* rats (Exp. 1) and Zebrafish (Exp. 2) ovarian tissue before (D0) and after in vitro culture (D1 and D7)

**Figure 4:**
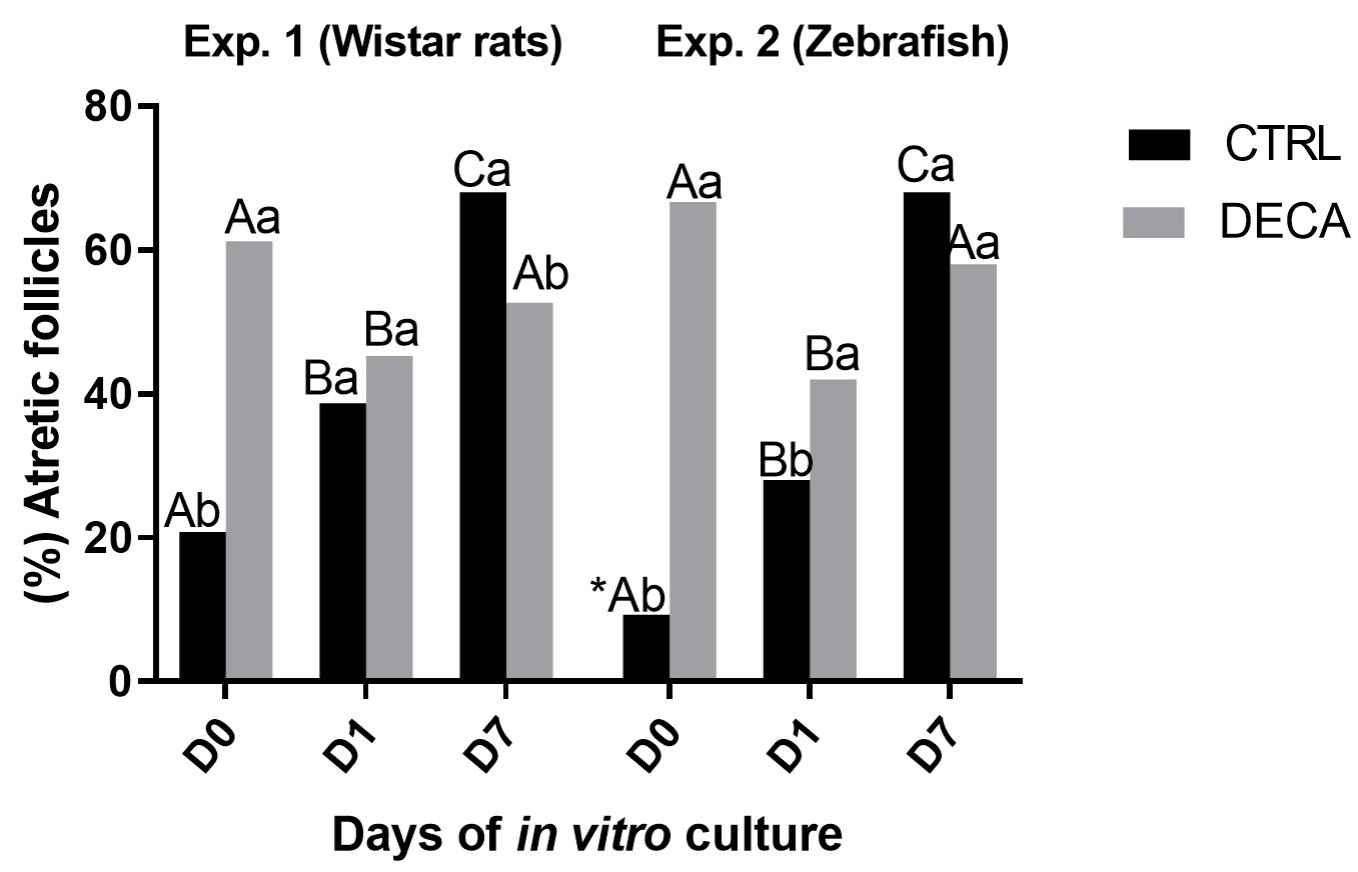
Percentage of atretic follicles (%) before and after 1 or 7 days of in vitro culture of ovaries from female *Wistar* rats (Experiment 1) and Zebrafish species (Experiment 2) after 7 weeks of treatment using only vehicle composed by peanut oil (CTRL group) or 10 mg/kg of Nandrolone decanoate (DECA group). *Indicate difference between the different animal species, within the same period and treatment, i.e, *Wistar* rats and Zebrafish in the Control (CTRLr D0 x CTRLz D0 or CTRLr D1 x CTRLz D1 or CTRLr D7 x CTRLz D7) or DECA (DECAr D0 x DECAz D0 or DECAr D1 x DECAz D1 or DECAr D7 x DECAz D7) (*P*<0.05). ^A,B^Indicate difference among days (D0, D1, D7) in CTRL or DECA group within the same animal species, i.e., *Wistar* rats (CTRLr D0 x CTRLr D1 x CTRLr D7 or DECAr D0 x DECAr D1 x DECAr D7) or Zebrafish (CTRLz D0 x CTRLz D1 x CTRLz D7 or DECAz D0 x DECAz D1 x DECAz D7) (*P*<0.05). ^a,b^Indicate difference between CTRL and DECA group within the same experimental days, i.e., D0, D1, D7 in *Wistar* rats (CTRLr D0 x DECAr D0 or CTRLr D1 x DECAr D1 or CTRLr D7 x DECAr D) or Zebrafish (CTRLz D0 x DECAz D0 or CTRLz D1 x DECAz D1 or CTRLz D7 x DECAz D) (*P*<0.05).

The percentage of atretic follicles can be observed in the Figure 4. It was demonstrated that in both experiments the number of atretic follicles was increased throughout the *in vitro* culture, from day 0 to day 7, in the Control group (CTRLr and CTRLz), while in the DECA group (DECAr and DECAz) the atretic follicles were reduced from D0 to D1 and increased over again on D7. Moreover, it is important to highlight that at D0, rat follicles were most harmed then Zebrafish follicles in control group (CTRLr vs CTRLz; P<0.05). At D7 the *in vitro* culture improved the rat ovary quality (Experiment 1), since the percentage of atretic follicles in the DECAr group was smaller (P<0.05) than CTRLr group. Regarding zebrafish follicles (Experiment 2), DECA exposure increase the atretic follicles on D0 and D1 when compared to CTRL group, except on D7 (DECAz vs CTRLz; P<0.05).

**Figure 5:**
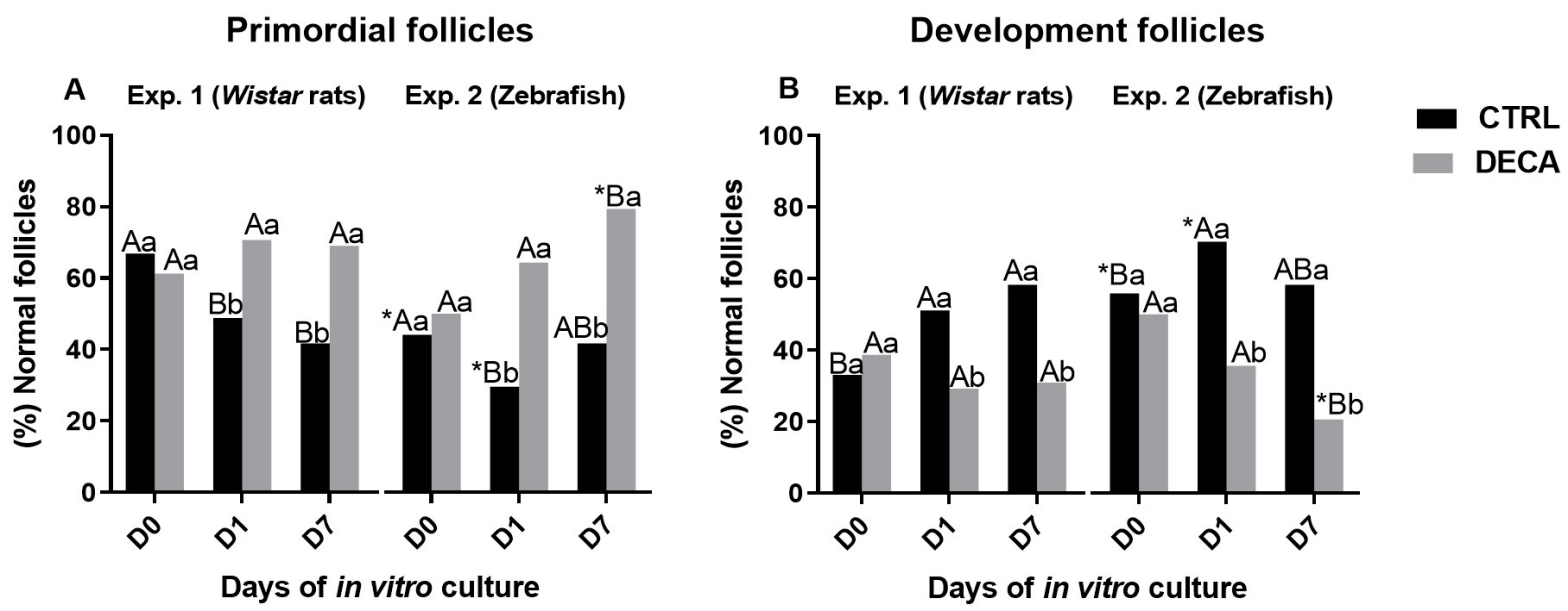
Percentage of primordial (A) or development follicles (B) of female animals of *Wistar* rats (Experiment 1) and Zebrafish species (Experiment 2) after 7 weeks of treatment using only vehicle composed by panut oil (CTRL group) or 10 mg/kg of Nandrolone decanoate (DECA group). *Indicate difference betwen the diferrents animal species, within the same period and tratamente, i.e, *Wistar* rats and Zebrafish in the Control (CTRLr D0 x CTRLz D0 or CTRLr D1 x CTRLz 1 or CTRLr D7 x CTRLz D7) or DECA (DECAr D0 x DECAz D0 or DECAr D1 x DECAz 1 or DECAr D7 x DECAz D7) (*P*<0.05). ^A,B^Indicate difference between of days (D0, D1, D7) CTRL or DECA group within the same animal species, i.e., *Wistar* rats (CTRLr D0 x CTRLr D1 x CTRLr D7 or DECAr D0 x DECAr D1 x DECAr D7) or Zebrafish (CTRLz D0 x CTRLz D1 x CTRLz D7 or DECAz D0 x DECAz D1 x DECAz D7) (*P*<0.05). ^a,b^Indicate difference between CTRL and DECA group within the same experimental days, i.e., *Wistar* rats D0, D1, D7 (CTRLr D0 x DECAr D0 or CTRLr D1 x DECAr D1 or CTRLr D7 x DECAr D), or Zebrafish (CTRLz D0 x DECAz D0 or CTRLz D1 x DECAz D1 or CTRLz D7 x DECAz D) (*P*<0.05).

Figure 5 demonstrate the percentage of primordial (Figure 5A) and development follicles (Figure 5B) in both experiments using *Wistar* rats (Experiment 1) or Zebrafish (Experiment 2). It was observed that for rats (Experiment 1) the primordial follicles activated and develop to antral follicles in CTRL group, i.e., the percentage of primordial follicles decrease (Figure 5A), while the percentage of development follicle increase (Figure 5B) from D0 to D7 (P<0.05). However, the exposure to DECA blocked follicle progression, i.e., there was no change in primordial or development follicle (P>0.05). Moreover, the percentage of development follicles was higher in CTRL then DECA group (P<0.05). Regarding the zebrafish ovaries, in CTRL group the percentage of primordial and development follicles reduced and increased, respectively, only from D0 to D1 (P<0.05), remaining constant on D7 (P>0.05). On the other hand, DECA exposure harmed follicle progression, since reduced the percentage of development follicle on D7 (P<0.05).

Comparing both species, Zebrafish increase the velocity of ovarian development, once the percentage of primordial follicles decrease (Figure 5A), while the percentage of development follicles increase (Figure 5B) compared to rat in D0 and D1 (P<0.05), becoming similar on D7 (P>0.05) in CTRL group (CTRLz vs CTRLr). However, to DECA groups the opposite happens, Zebrafish ovaries (DECAz) have a percentage of primordial and development follicles, respectively higher and lower on D7 (P<0.05), when compared to rats (DECAr), which characterize the impairment of follicular progression.

### Redox status by thiol content, and SOD and CAT activity measurements

**Figure 6:**
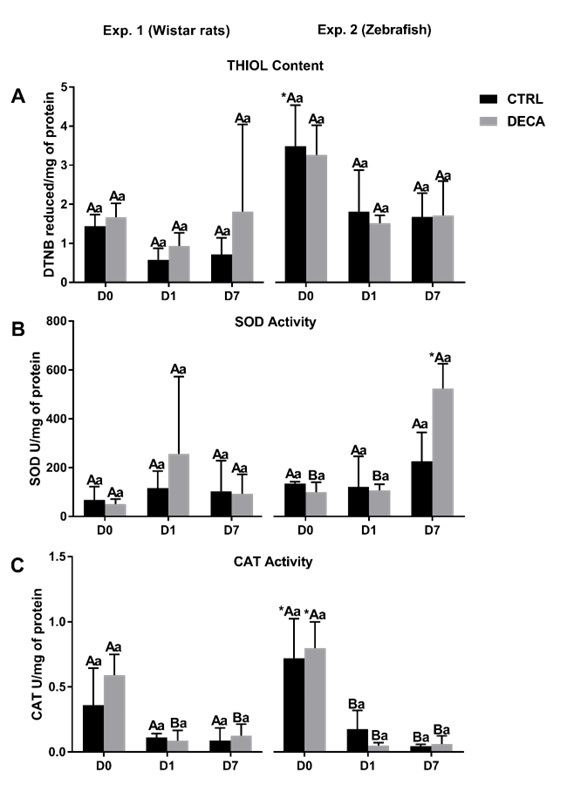
Redox status, measured as (A) Thiol content, (B) superoxide dismutase (SOD), and (C) catalase (CAT) in female animals of *Wistar* rats (Experiment 1) and Zebrafish species (Experiment 2) after 7 weeks of treatment using only vehicle composed by peanut oil (CTRL group) or 10 mg/kg of Nandrolone decanoate (DECA group). Values are expressed in mean ± s.e.m. *Indicate difference between the different animal species, within the same period and treatment, i.e, *Wistar* rats and Zebrafish in the Control (CTRLr D0 x CTRLz D0 or CTRLr D1 x CTRLz 1 or CTRLr D7 x CTRLz D7) or DECA (DECAr D0 x DECAz D0 or DECAr D1 x DECAz 1 or DECAr D7 x DECAz D7). ^A,B^Indicate difference between of days (D0, D1, D7) CTRL or DECA group within the same animal species, i.e., *Wistar* rats (CTRLr D0 x CTRLr D1 x CTRLr D7 or DECAr D0 x DECAr D1 x DECAr D7) or Zebrafish (CTRLz D0 x CTRLz D1 x CTRLz D7 or DECAz D0 x DECAz D1 x DECAz D7) (P<0.05). ^a,b^Indicate difference between CTRL and DECA group within the same experimental days, i.e., *Wistar* rats D0, D1, D7 (CTRLr D0 x DECAr D0 or CTRLr D1 x DECAr D1 or CTRLr D7 x DECAr D), or Zebrafish (CTRLz D0 x DECAz D0 or CTRLz D1 x DECAz D1 or CTRLz D7 x DECAz D) (*P*<0.05).

Thiol content, and SOD and CAT activity in ovarian tissue of *Wistar* rats (Exp. 1) and Zebrafish females (Exp. 2) is shown in Figure 6. The antioxidant environment represented by the increase of thiol content (Figure 6A) was significantly (P<0.05) higher in the D0 of CTRLz than CTRLr. Regarding the SOD activity (Figure 6B), it was observed that, while there is no difference in the *Wistar* rats groups, in Zebrafish groups occurs an increase of this enzyme activity from D0 to D7. Moreover, the SOD activity is also increased (P<0.05) at D7 in the Zebra fish group exposed to DECA (DECAz), when compared to *Wistar* rats groups (DECAr). Regarding the CAT activity (Figure 6C) at D0, Control and DECA groups from Exp. 2 were higher than Exp. 1 (CTRLr versus CTRLz or DECAr versus DECAz). When compared the days of culture, it was observed that from D0 to D7 in both models, *Wistar* rats (Exp. 1) and Zebrafish (Exp. 2), DECA exposure decreased (P<0.05) CAT activity. However, only control group from Exp. 1 (CTRLr) maintained the CAT activity from D0 similar to D7 (P>0.05).

## Discussion

To our knowledge this is the first study to directly compare Zebrafish and *Wistar* rats, affirming that Zebrafish can be used as experimental model to other mammalian species, including to human beings. Moreover, it was the first time that the effects of *in vitro* culture of ovarian tissue of Zebrafish and *Wistar* rats treated with an anabolic steroid during 7 weeks were evaluated. In both experiments, DECA exposition have changed the body weight from initial to final experimental period, which was increased in *Wistar* rats (Exp. 1) and decreased in Zebrafish (Exp. 2). Similar to our findings, to treat Zebrafish with testosterone, Lee and Renshaw (2017) verified a decrease in the gonadosomatic index, i.e, body and gonad weight reduction, besides a libido reduction and an interruption in follicle development, with reduction of mature oocyte number.

Considering that a failure in follicular development would cause a reduction in circulating estradiol levels, that would explain the disrupted of the estrous cycle in, keeping the animals in the progestogen phase in both groups treated with DECA (DECAr and DECAz). In addition, we have demonstrated that the number of atretic follicles was increased and development follicles were decreased, which could impair estradiol production. Similar to our results in *Wistar* rats, Simão et al. (2015) verified that the body weight gains and estrous cycle interruption by DECA. This effect may be due to its anabolic characteristic, promoting muscle fiber hypertrophy and increase of protein synthesis (Shahidi, 2001).

As can be observed in the water characteristics of the Experiment 2, in which the Zebrafish was used as an experimental model, the design of this study strictly followed and respected all the recommendations of a Brazilian Resolution N^o^ 34/2017 of National Council for the Control of Animal Experimentation (CONCEA), ensuring the suitable conditions to use these animals for teaching and research (Brasil, 2017). As well as food and nutrition, water quality has a major impact on Zebrafish general health, including in their reproduction physiology (Harper and Lawrence, 2016). The exposure to anabolic steroids did not alter the water quality parameters for the exposed animals. The oxygen levels reduction during exposure remained adequate according to the conditions established for zebrafish maintenance, which is 4 mg/L (Westerfield, 2007). In relation to the ammonia and nitrite levels, their levels were low before exposure and zero during exposure to anabolic steroids. It is important to note that ammonia and nitrite are result of animal excreta and can be produced during the decomposition of organic matter in the aquarium water. Thus, we can affirm that Zebrafish were properly housed and handled by our researcher team, ensuring the reliability of the data.

Similar to Simão et al. (2015), in the present study the treatment with DECA kept female *Wistar* rats in progestogen phase of the estrous cycle. In other study, the same team observed that 15 mg/Kg of DECA caused the animals to remain in diestrus by the hypersecretion of luteinizing hormone (LH) (Gervasio et al., 2014) and hyposecretion of gonadal hormones (estrogen and progesterone) (Belardin et al., 2014). On the other hand, in our experiment it is possible to infer that *in vitro* culture could reverse DECA effects, since the percentage of atretic follicles have decreased on day 7, even though this reversal has not yet been observed *in vivo* by cessation of androgen administration.

Similar to Souza et al. (2018), in our experiment the reduction of health follicles and consequently increase of atretic follicles after DECA exposition, could promotes hormonal imbalance and ovulation failure (Dixon et al., 2014; Simão et al., 2016). As in *Wistar* rats, we observed that the exposure to DECA also caused an increase in the percentage of atretic follicles in Zebrafish and impaired follicle development. However, the *in vitro* culture was able to reverse this deleterious effect, since there was a reduction in the percentage of degenerated follicles at the end of the in vitro culture (D7) in both models, increasing the percentage of primordial follicles in Zebrafish at D7. Corroborating with our results, and in a complementary way, Lee and Renshaw (2017) and Faltermann et al. (2020) observed that Zebrafish exposure to steroids androgenic caused the interruption of follicular development. This can be due the fact of vitellogenin production by the Zebrafish liver would also be reduced, leading to losses in the yolk production by the ovarian follicles (Faltermann et al., 2020). Moreover, zebrafish lost body weight after DECA administration. Consequently, all these effects may cause gonadal atrophy and a decrease in the number of mature follicles. However, as mentioned before, primordial follicles increase in zebrafish. We believe that it can be due the fact that primordial germinal cells in Zebrafish continue to proliferate and differentiate in oogonia, renewing the oocyte stock (Tokarz, 1978; Grier and Lo Nostro, 2000). These differences between the studded experimental species may be due to a possible oxidative stress, since that SOD activity was increased and CAT activity was decreased from D0 to D7 in Zebrafish groups, which was not observed to *Wistar* rats groups.

The impacts observed in both models in the present study suggest that the Zebrafish model could be considered as an option of experimental model for other mammalian species, including human being. According to Silva (2015), studies on reproductive characteristics and the use of techniques and tools for animal reproduction have advanced significantly. Although in vitro culture is widely used as a tool to assess ovarian physiology in cattle, goats, and horses, further research is needed to apply this technique in fish (Silva, 2015). This would help refine methodologies aimed to increase reproductive success in fish, as some fish species are declining in the wild. Additionally, it promotes their use as an alternative model to mammals, particularly as a biological model to optimize studies focused on human reproduction, given that fish development is fast and cheap compared to others mammalian models.

## Conclusion

In conclusion, the present study demonstrated similarity in ovarian physiology between *Wistar* rats and Zebrafish exposed or not to anabolic steroid Nandrolone decanoate (DECA); then, we propose the substitution, when convenient. Furthermore, in vitro culture proved to be an excellent tool for reversing some of the deleterious effects caused by DECA on the ovaries of female mammals. However, it still needs more studies to better understand ovarian physiology similarities between fish and mammalian models.
